# The Antimetastatic Effects of Resveratrol on Hepatocellular Carcinoma through the Downregulation of a Metastasis-Associated Protease by SP-1 Modulation

**DOI:** 10.1371/journal.pone.0056661

**Published:** 2013-02-20

**Authors:** Chao-Bin Yeh, Ming-Ju Hsieh, Chiao-Wen Lin, Hui-Ling Chiou, Pen-Yuan Lin, Tzy-Yen Chen, Shun-Fa Yang

**Affiliations:** 1 School of Medicine, Chung Shan Medical University, Taichung, Taiwan; 2 Department of Emergency Medicine, Chung Shan Medical University, Taichung, Taiwan; 3 Department of Emergency Medicine, Chung Shan Medical University Hospital, Taichung, Taiwan; 4 School of Medical Laboratory and Biotechnology, Chung Shan Medical University, Taichung, Taiwan; 5 Institute of Oral Sciences, Chung Shan Medical University, Taichung, Taiwan; 6 School of Pharmacy, Taipei Medical University, Taipei, Taiwan; 7 Department of Internal Medicine, Chung Shan Medical University Hospital, Taichung, Taiwan; 8 Institute of Medicine, Chung Shan Medical University, Taichung, Taiwan; 9 Department of Medical Research, Chung Shan Medical University Hospital, Taichung, Taiwan; The University of Kansas Medical Center, United States of America

## Abstract

**Background:**

The mortality and morbidity rates from cancer metastasis have not declined in Taiwan, especially because of hepatocellular carcinoma (HCC). Resveratrol has been shown to have benefits such as cardioprotection, providing antioxidative, anti-inflammatory, anti-cancer properties in previous studies. Therefore, HCC cells were subjected to treatment with resveratrol and then analyzed to determine the effects of resveratrol on the migration and invasion.

**Methodology and Principal Findings:**

Modified Boyden chamber assays revealed that resveratrol treatment significantly inhibited cell migration and invasion capacities of Huh7 cell lines that have low cytotoxicity in vitro, even at a high concentration of 100 µM. The results of casein zymography and western blotting revealed that the activities and protein levels of the urokinase-type plasminogen activator (u-PA) were inhibited by resveratrol. Western blot analysis also showed that resveratrol inhibits phosphorylation of JNK1/2. Tests of the mRNA level, real-time PCR, and promoter assays evaluated the inhibitory effects of resveratrol on u-PA expression in HCC cells. The chromatin immunoprecipitation (ChIP) assay showed that reactive in transcription protein of nuclear factor SP-1 was inhibited by resveratrol.

**Conclusions:**

Resveratrol inhibits u-PA expression and the metastasis of HCC cells and is a powerful chemopreventive agent. The inhibitory effects were associated with the downregulation of the transcription factors of SP-1 signaling pathways.

## Introduction

Hepatocellular carcinoma (HCC) is a common malignant neoplasm and cancer-related death in Asian countries. The mortality rate of HCC in Taiwan has not decreased principally because of uncontrolled tumor invasion and metastasis [1]. The metastasis of cancer cells typically involves multiple processes, including the invasion of surrounding tissue, penetration into blood or lymphatic vessels, and the formation of new tumors (i.e., moving from the primary to the secondary site) [2]. The first critical cytophysiological changes that occur during metastasis include altered adhesive capabilities between cells, extracellular matrix (ECM) with proteolytic degradation, and the damaging of intercellular interactions. The degradation of ECM by cancer cells through protease, such as serine proteinase, matrix metalloproteinases (MMPs), cathepsins, and plasminogen activator (PA), may cause the separation of the intercellular matrix, promoting the mobility of cancer cells and eventually leading to metastasis [3]. Among these involved proteases urokinase-type plasminogen activator (u-PA) is the most important degradations to the basement membrane and is prominently involved in tumor invasion and metastasis [4]. Pathological states including cancer, inflammation, and vascular diseases could increase proteinase activity. u-PA is a serine protease involved in ECM degradation, invasion, and metastasis by cancer cells because it regulates the plasminogen/plasmin system. The u-PA applies its effect by binding to the u-PA receptor (u-PAR) and localizing on the cell surface of u-PA and enhancing its plasminogen activation capability effect. This activity is negatively regulated by plasminogen activator inhibitor types 1 and 2 (PAI-1 and -2). The imbalance between u-PA and PAIs may contribute to the degradation or deposition of ECM [5]. Therefore, inhibiting the migration or invasion mediated by u-PA could prevent cancer metastasis.

Resveratrol (C14H12O3; 3,4′,5-trihydroxystilebene) was originally isolated from the roots of white hellebore by Takaoka in 1940 [6]. Resveratrol belongs to the stilbene group and is a main component of wine [7], [8]. Resveratrol has been used in traditional Japanese and Chinese medicine to treat fungal diseases, various skin inflammations, and cardiovascular and liver diseases [9], [10]. Resveratrol has recently been shown to have various therapeutic purposes, including antioxidation, anti-proliferation, and chemopreventive effects [11], [12]. Additionally, accumulating evidence indicates that resveratrol possesses an antitumor effect by inhibiting tumor cell growth and inducing apoptosis [13]–[16]. However, limited studies exist concerning the anti-metastasis effects of resveratrol. The present study aimed to investigate the effects of resveratrol on cell migration and invasion in cultured hepatocellular carcinoma and to study the possible underlying mechanisms.

## Materials and Methods

### Cell Culture and Resveratrol Treatment

HCC (Huh7) cells obtained from the Food Industry Research and Development Institute (Hsinchu, Taiwan) were cultured using Dulbecco’s modified Eagle’s medium (Life Technologies, Grand Island, NY, USA) containing 10% fetal bovine serum, 2 mM glutamine, 100 U/ml penicillin, 100 µg/ml streptomycin, and 400 ng/ml hydrocortisone. All cell cultures were maintained at 37°C in a humidified atmosphere of 5% CO2. For resveratrol treatment, an appropriate concentration of resveratrol (Sigma chemical Co., St. Louis, MO, USA) was added to the culture medium to achieve the indicated concentrations and then incubated with cells for the indicated time periods. A dimethylsulfoxide solution without resveratrol was used as the blank reagent.

### The Determination of Cell Viability (MTT Assay)

For the cell viability experiment, a microculture tetrazolium (3-(4,5-dimethylthiazol-2-yl)-2,5-diphenyltetrazolium bromide) colorimetric assay was performed to determine the cytotoxicity of resveratrol [17]. Huh-7 cells were seeded in 24-well plates at a density of 5×10^4^ cells/well and were treated with resveratrol at concentrations ranging from 0 to 200 µM at 37°C for 24 h. Following the exposure period, the medium was removed and the cells were washed with phosphate buffered saline (PBS) and incubated with 20 µl MTT (5 mg/ml) (Sigma chemical Co., St. Louis, MO, USA) for 4 h. The viable cell number per dish was directly proportional to the production of formazan, which was measured spectrophotometrically at 563 nm following solubilization using isopropanol.

### In Vitro Wound Closure

Huh7 cells (1×10^5^ cells/well) were plated in 6-well plates for 24 h, wounded by scratching with a pipette tip, incubated with a DMEM medium containing 0.5% FBS, and treated with or without resveratrol (0, 25, 50, 75 and 100 µM) for 0, 12, and 24 h. The cells were photographed using a phase-contrast microscope (×100).

### Cell Invasion and Migration Assays

Cell invasion and migration were assayed according to the methods described by Yang et al [3]. After treatment with resveratrol (0, 25, 50, 75 and 100 µM) for 24 h, surviving cells were harvested and seeded in a Boyden chamber (Neuro Probe, Cabin John, MD, USA) at 10^4^ cells/well in a serum-free medium and then incubated for 24 h at 37°C. For the invasion assay, 10 µl Matrigel (25 mg/50 ml; BD Biosciences, MA, USA) were applied to polycarbonate membrane filters with 8 µm pore size; the bottom chamber contained a standard medium. The filters were then air dried for 5 h in a laminar flow hood. The invaded cells were fixed with 100% methanol and stained with 5% Giemsa. The cell numbers were counted using a light microscope. The migration assay was conducted as described in the invasion assay with no coating of the matrigel [3].

### Determination of u-PA by Zymography

The activities of u-PA in a conditional medium were measured using casein zymography protease assays as previously described [18]. Briefly, the collected media of an appropriate volume (adjusted according to vital cell number) were prepared using an SDS sample buffer without boiling or reduction and subjected to 2% casein (w/v) and 20 µg/ml plasminogen-8% SDS-PAGE electrophoresis. After electrophoresis, the gels were washed with 2.5% Triton X-100 and incubated in a reaction buffer (40 mM Tris–HCl, pH 8.0; 10 mM CaCl2; and 0.01% NaN3) for 12 h at 37°C. Thereafter, the gel was stained with Coomassie brilliant blue R-250.

### RNA Preparation and TaqMan Quantitative Real-time PCR

The total RNA was isolated from HCC cells using Trizol (Life Technologies, Grand Island, NY, USA) according to the manufacturer’s instructions. Quantitative real-time PCR analysis was performed using a Taqman one-step PCR Master Mix (Applied Biosystems). In total, 100 ng of cDNA was added per 25 µl reaction with u-PA or GAPDH primers and TaqMan probes. Quantitative real-time PCR assays were performed in triplicate on a StepOnePlus sequence detection system. The oligonucleotide sequences of TaqMan probes and primers were described in Table 1. The threshold was set above the non-template control background and within the linear phase of the target gene amplification to determine the cycle number at which the transcript was detected.

**Table 1:** Primers list for real-time PCR and ChIP assay.

Primers used inreal-time PCR	Sequence (5′ to 3′)
u-PA (Hs01547054_m1)	(FAM)-CAACGACATTGCCTTGCTGAAGATC
GAPDH (Hs99999905_m1)	(FAM)-GGCGCCTGGTCACCAGGGCTGCTTT
Primers used in ChIP	
u-PA SP-1-F	CAGGTGCATGGGAGGAAGC
u-PA SP-1-R	AGGGGCGGCGCCGGGGCGG

### Preparation of Total Cell Lysates and Nuclear Fraction

For preparation of the total cell lysates, the cells were rinsed with PBS twice, scraped with 0.2 ml of a cold RIPA buffer containing a protease inhibitors cocktail, and then vortexed at 4°C for 10 min. Thereafter, the cell lysates were subjected to a centrifugation of 10000 rpm for 10 min at 4°C and the insoluble pellet was discarded. The nuclear extracts were obtained using a modification of a previously described method [19]. Briefly, the harvested cells were scraped and lysed with Buffer A (10 mM HEPES, 10 mM KC1, 0.1 mM EDTA, 1.5 mM MgCl2, 0.2% NP40, 1 mM DTT, and 0.5 mM phenylmethylsulfonyl fluoride), vortexed to shear the cytoplasmic membranes, and then the nuclear pellets were collected using centrifugation at 3000 rpm for 30 s at 4°C. The nuclear proteins were extracted using high-salt Buffer B (20 mM HEPES, 25% glycerol, 1.5 mM MgCl2, 0.1 mM EDTA, 420 mM NaCl, 1 mM DTT, and 0.5 mM phenylmethylsulfonyl fluoride). The protein concentration of the total cell lysates and the nuclear fraction were determined by Bradford assay [20].

### Western Blot Analysis

The cell lysates and nuclear extracts were separated in a 10% polyacrylamide gel and transferred onto a nitrocellulose membrane. The blot was subsequently incubated with 5% non-fat milk in Tris-buffered saline (20 mM Tris, 137 mM NaCl, pH 7.6) for 1 h to block non-specific binding and then overnight with polyclonal antibodies against u-PA, SP-1, C23 (internal control for nuclear), or with the specific antibodies for unphosphorylated or phosphorylated activated forms of the corresponding ERK 1/2, JNK 1/2, p38, and Akt. The blots were then incubated using a horseradish peroxidase goat and anti-rabbit or anti-mouse IgG for 1 h. Thereafter, the signal was detected using an enhanced chemiluminescence (ECL) commercial kit (Amersham Biosciences), and the relative photographic density was quantitated by scanning the photographic negatives on a gel documentation and analysis system (AlphaImager 2000, Alpha Innotech Corporation, San Leandro, CA, USA).

### Transfection and u-PA Promoter-driven Luciferase Assays

Huh7 cells were seeded at a concentration of 5×10^4^ cells per well in 6-well cell culture plates. After 24 h of incubation, the pGL3-basic (vector) and the u-PA promoter plasmid were co-transfected with a β-galactosidase expression vector (pCH110) into cells using Turbofect (Fermentas, Carlsbad, CA, USA). After 12 h of transfection, the cells were treated with a vehicle or resveratrol (0, 25, 50, 75 and 100 µM) for 24 h. The cell lysates were harvested and the luciferase activity was determined using a luciferase assay kit. The value of the luciferase activity was normalized to transfection efficiency and monitored by β-galactosidase expression.

### Chromatin Immunoprecipitation Analysis (ChIP)

Chromatin immunoprecipitation analysis was performed as described previously [21]. The DNA was immunoprecipitated with anti-SP-1. The anti-SP-1 was purified and extracted using phenol-chloroform. The immunoprecipitated DNA was analyzed using PCR or quantitative PCR using specific primers as described in Table 1.

### Statistical Analysis

The statistical significances of the differences throughout this study were analyzed using one-way ANOVA to compare the differences between treatments; follow-up was performed using Dunnett’s multiple comparison post hoc test. A difference of *p*<0.05 was considered statistically significant, and all experiments were repeated 3 times.

## Results

### The Effect of Resveratrol on the Viability of Huh7 Cells and Normal Hepatocytes

**Figure 1:**
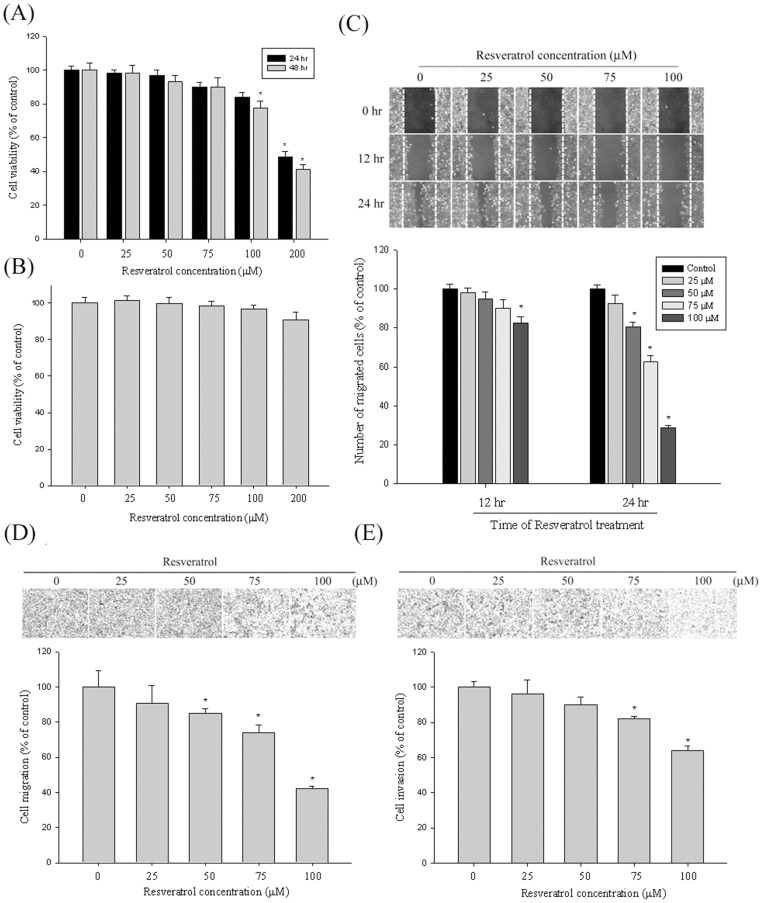
The effect of resveratrol on cell viability, in vitro wound closure, and cell migration and invasion in Huh7 cells. (A) Huh7 cells were treated with resveratrol (0, 25, 50, 75, 100 and 200 µM) for 24 h and 48 h before being subjected to a MTT assay that tested cell viability. The values represented the means ± SD of at least 3 independent experiments. (B) Normal hepatocytes were treated with resveratrol (0, 25, 50, 75, 100 and 200 µM) for 24 h before being subjected to a MTT assay that tested cell viability. The values represented the means ± SD of at least 3 independent experiments. (C) Huh7 cells were wounded and then treated with vehicle (DMSO) or resveratrol (25, 50, 75, and 100 µM) for 0, 12, and 24 h in a 0.5% FBS-containing medium. At 0, 12, and 24 h, the phase-contrast pictures of the wounds at 3 locations were taken. (D & E) Cell migration and invasion were measured using a Boyden chamber for 16 and 24 h with polycarbonate filters, respectively. The migration and invasion abilities of Huh7 cells were quantified by counting the number of cells that invaded the underside of the porous polycarbonate, as described in the Materials and Methods section. The values represented the means ± SD of at least 3 independent experiments. *p<0.05, compared with the vehicle group.

The cytotoxic effects of resveratrol at various concentrations (0–200 µM) on Huh7 cells for 24 and 48 hr are shown in Fig. 1A. The MTT assay showed that, at the highest concentration of 200 µM, resveratrol altered HCC cell viability (p<0.05). Therefore, we used a lower concentration range of resveratrol for all subsequent experiments. Furthermore, resveratrol did not alter the cell viability of normal hepatocytes (p>0.05) (Fig. 1B).

### The Effects of Resveratrol on in vitro Wound Closure, Invasion, and Migration in Huh7 Cells

Figure 1C shows the findings from a wound closure assay that determined the effects of resveratrol on the migration of Huh7 cells and contains representative photographs of Huh7 cells migrating into scratch wounds during treatment using resveratrol. At 100 µM, resveratrol decreased the migrated cell number by 18% and 82% at 12 h, and 24 h, respectively. Figures 1D and 1E show the effect of resveratrol on cell migration and invasion in Huh7 cells that were treated with 0, 25, 50, 75, and 100 µM of resveratrol for 16 h (cell migration) and 24 h (cell invasion). We showed that resveratrol reduced the migration and invasion of Huh7 cells substantially in a concentration-dependent manner (p<0.05).

### The Effect of Resveratrol on the Protein Levels of u-PA and their Endogenous Inhibitors

**Figure 2:**
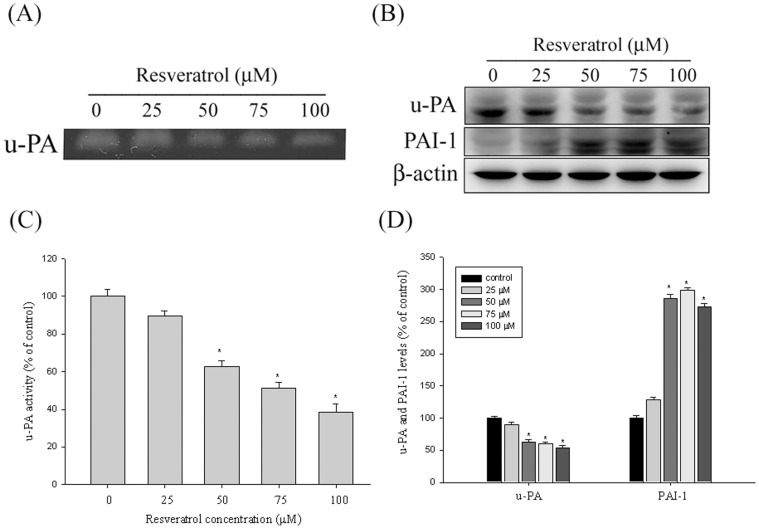
Effects of resveratrol on the activity and protein level of u-PA and the protein level of the endogenous inhibitor PAI-1. (A, C) Huh7 cells were treated with resveratrol (0, 25, 50, 75, and 100 µM) for 24 h and then subjected to casein zymography to analyze the activity of the u-PA. (B, D) Huh7 cells were treated with resveratrol (0, 25, 50, 75, and 100 µM) for 24 h and then subjected to western blotting to analyze the protein levels of u-PA and PAI-1. The quantitative results of u-PA and PAI-1 protein levels that were adjusted with the β-actin protein level. The values represented the means ± SD of at least 3 independent experiments. *p<0.05, compared to the vehicle group.

Huh7 cells were treated with resveratrol (0, 25, 50, 75, and 100 µM) for 24 h, and thereafter, were subjected to casein zymography to analyze u-PA activity. As shown in Figs. 2A and 2C, resveratrol treatment reduced the activity and protein levels of u-PA in a dose-dependent manner. Figure 2B shows western blotting analysis of the protein levels of u-PA and PAI-1. The u-PA and PAI-1 protein levels were adjusted using β-actin. The protein levels of u-PA decreased significantly, whereas those of their respective PAI-1 increased (Figure 2D).

### The Effect of Resveratrol on the Transcriptional Level of u-PA

**Figure 3:**
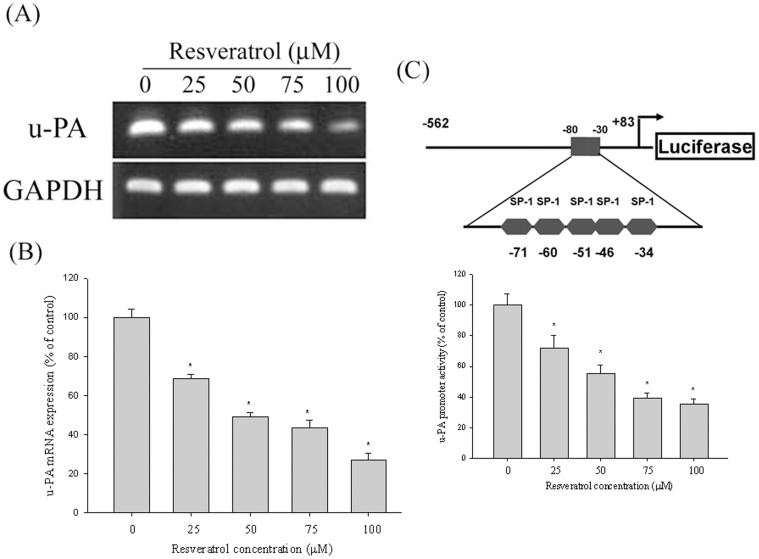
Resveratrol suppresses u-PA expression at a transcriptional level. Huh7 cells were treated with resveratrol (0, 25, 50, 75, and 100 µM) for 24 h and then subjected to (A) reverse-transcription PCR and (B) quantitative real-time PCR to analyze the mRNA expression of u-PA. (C) u-PA promoter reporter assay to analyze the promoter activity of u-PA. Luciferase activity, determined in triplicate, was normalized to β-galactosidase activity. The values represented the means ± SD of at least 3 independent experiments. *p<0.05, compared with the vehicle group.

Tests of the mRNA and the real-time PCR and promoter reporter assays evaluated the inhibitory effects of resveratrol on u-PA expression in Huh7 cells. The Huh7 cells were treated with 0, 25, 50, 75, and 100 µM resveratrol for 24 h and were then subjected to RT-PCR and real-time PCR to analyze mRNA levels. The u-PA mRNA levels decreased considerably in a concentration-dependent manner following treatment with various concentrations of resveratrol (Figures 3A and 3B). As shown in Figure 3C, the luciferase activities of u-PA were significantly suppressed, as determined using a luciferase assay kit. These results show that resveratrol regulates u-PA expression, at least partially, at a transcriptional level.

### SP-1 is the Key Regulator for the Transcriptional Inhibition of u-PA Using Resveratrol

**Figure 4:**
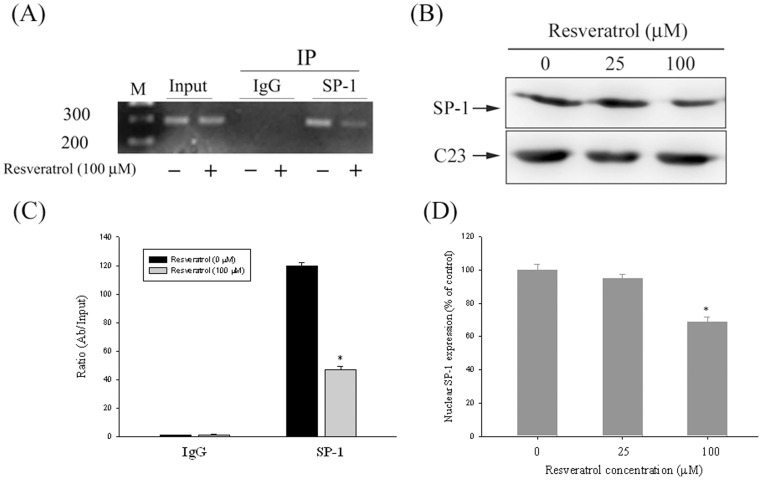
Critical role of SP-1 in resveratrol -induced transcriptional inhibition of u-PA in Huh7 cells. (A & C) Huh7 cells were treated with resveratrol (0 and 100 µM) for 24 h and then the nuclear fraction was prepared as described in Materials and Methods section. (B) Levels of SP-1 in the nucleus that was immunodetected with SP-1 specific antibodies. Representative results of SP-1 protein levels by western blot analysis. (D) Quantitative results of SP-1 protein levels that were adjusted with the C23 protein level. The values represented the means ± SD of at least 3 independent experiments. *p<0.05, compared with the vehicle group.

Sequence analysis of the u-PA promoter revealed the cis-acting regulatory elements, SP-1, which could be involved in the regulation of u-PA expression. We performed a chromatin immunoprecipitation (ChIP) assay to investigate the influence that transcription factors have on the transcriptional inhibitory effects of resveratrol on u-PA (Figure 4A). Quantitative real-time PCR assay showed that resveratrol substantially suppressed the binding of SP-1 to the u-PA promoters (Figure 4C). To further test whether SP-1 is involved in the transcriptional regulation of resveratrol on u-PA in Huh7 cells, we evaluated the effect of resveratrol on the nuclear translocation of SP-1. Treating Huh7 cells with 0, 25, and 100 µM of resveratrol reduced the nuclear translocation of SP-1 (Figures 4B and 4D). The findings indicate that resveratrol might induce transcriptional inhibition of u-PA in Huh7 cells by suppressing SP-1 nuclear translocation and u-PA promoter binding activity.

### The Effect of Resveratrol on MAPK and PI3K/Akt Pathways

**Figure 5:**
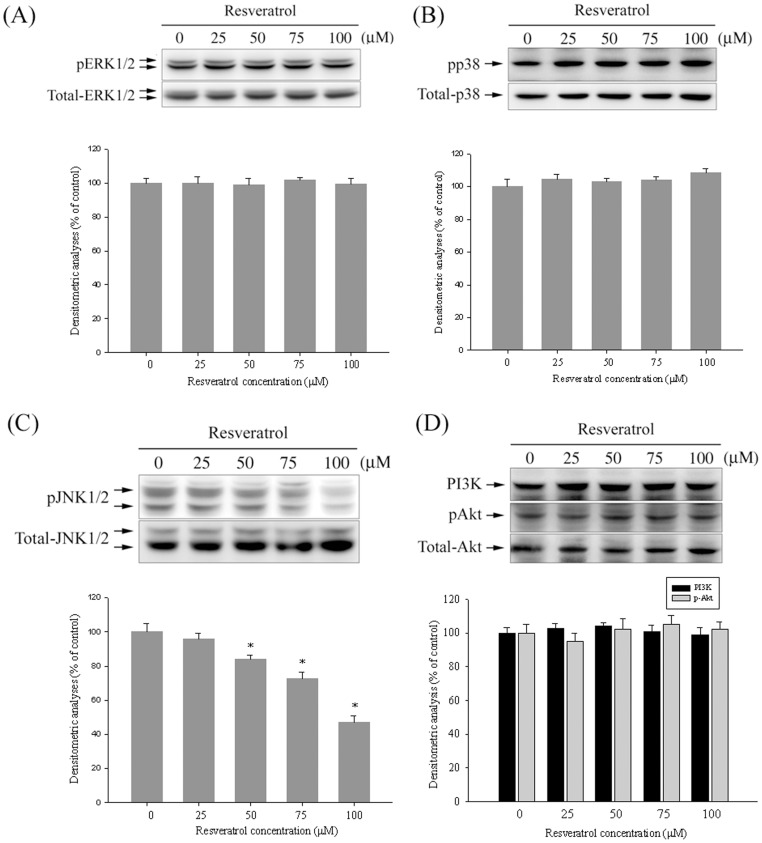
Effects of resveratrol on the MAPKs pathway and PI3K/Akt signalings. Huh7 cells were cultured in various concentrations of resveratrol (0, 25, 50, 75 and 100 µM) for 24 hours, and then the cell lysates were subjected to SDS–PAGE followed by western blots with (A) anti-ERK1/2, (B) anti-p38, (C) anti-JNK and (D) anti-PI3K and anti-Akt (total and phosphorylated) antibodies as described in Materials and Methods. Determined activities of these proteins were subsequently quantified by densitometric analyses with that of control being 100% as shown just below the gel data. The values represented the means ± SD of at least 3 independent experiments. *p<0.05 as compared with the vehicle group.

After the inhibitory effect of resveratrol on the cell migration/invasion and proteinases was revealed, the effects of resveratrol on the expressions of MAPK and PI3K/Akt pathways were investigated using western blotting to elucidate their underlying mechanisms. Western blotting showed that resveratrol reduced the phosphorylation of JNK 1/2 (Figure 5C) in Huh7 cells. According to densitometric analyses of blots versus the control, treatment of resveratrol at 100 µM resulted in a reduction in the phosphorylation of JNK 1/2 to 46%. However, the phosphorylation of the ERK1/2, p38, and PI3K/Akt pathways remained unaffected (Figures 5A, B and D).

### The Effect of Resveratrol on u-PA Expression, in vitro Wound Closure, and the Migration and Invasion of HCC Cells with SP600125

**Figure 6:**
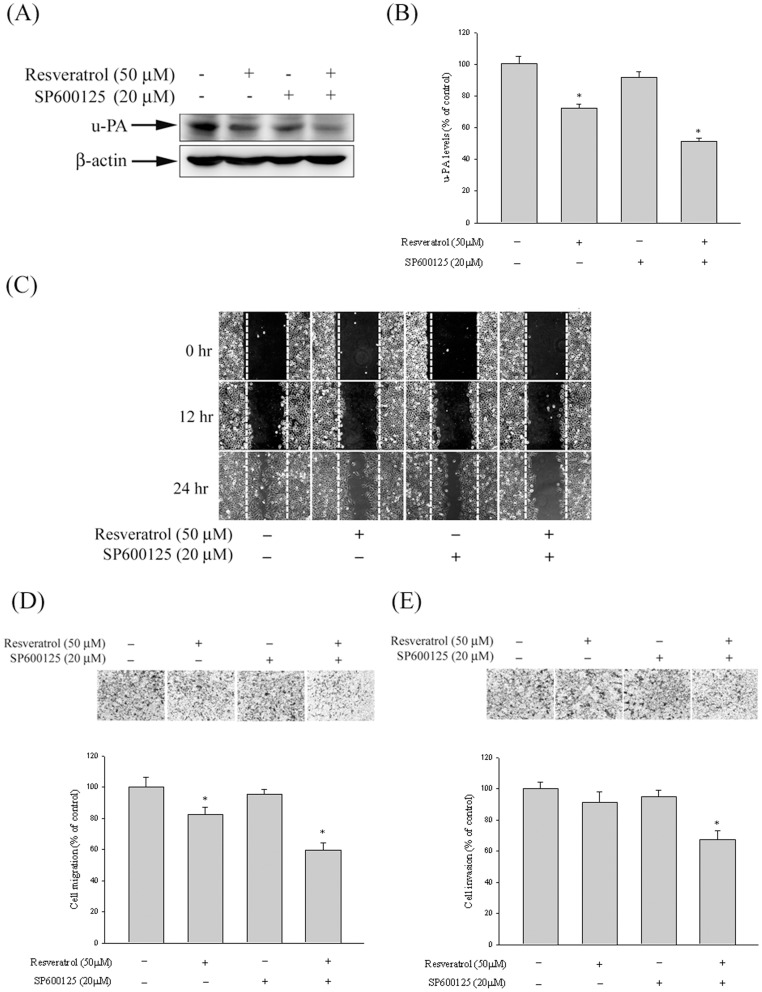
Effect of resveratrol and JNK inhibitor (SP600125) on u-PA expression, *in vitro* wound closure, cell migration and invasion in Huh7 cells. (A–B) Huh7 cells were pre-treated with SP600125 for 30 min and then incubated in the presence or absence of resveratrol for 24 h, and then the cell lysates were subjected to SDS–PAGE followed by western blots with anti-u-PA antibodies as described in Materials and Methods. (C–E) Huh7 cells were pre-treated with SP600125 for 30 min and then incubated in the presence or absence of resveratrol for 24, Huh7 cells were then subjected to in vitro wound closure, cell migration and invasion assay. The migration and invasion abilities of Huh7 cells were quantified by counting the number of cells that invaded to the underside of the porous polycarbonate as described in the Materials and Methods section. The values represented the means ± SD of at least three independent experiments. *p<0.05 as compared with the vehicle group.

To further determine whether resveratrol inhibition of proteinase, invasion, and migration was caused mainly by inhibiting the JNK 1/2 signaling pathway, we investigated its effects on a specific inhibitor of the JNK 1/2 pathway (SP600125) in Huh7 cells. The results showed that a combined treatment of the inhibitor and resveratrol further reduced u-PA expression (Figures 6A and 6B). In addition, we observed a similar trend for the inhibition of Huh7 migration and invasion for the single and combined treatments (Figures 6C to 6E). Therefore, the inhibition of the JNK 1/2 signaling pathways may result in a reduced expression of u-PA, as well as reduced tumor cell invasion.

## Discussion

The clinical usefulness of herbal products in the prevention and treatment of various chronic diseases and cancers has been globally recognized. Liu et al. revealed that resveratrol inhibits mammalian target of rapamycin (mTOR) signaling when used in therapy for cardiovascular and metabolic disorders [22]. However, most studies have focused on resveratrol’s anticancer effects, such as apoptosis. Resveratrol has several therapeutic effects on cancer cells, including the inhibition of cell proliferation, anti-angiogenic effects, cell-cycle blockage, and the induction of cell apoptosis [8], [23], [24]. Therefore, resveratrol has been used for the treatment of primary hepatoma, lung cancer, breast cancer, and colon cancer [24]–[27]. However, the mechanism of how resveratrol affects pathways to cause anti-metastasis remains unclear, especially in HCC cells. This study verifies that resveratrol inhibits u-PA through phosphorylation of the JNK 1/2 pathway to induce an anti-metastasis effect.

The metastasizing of cancer cells typically involves multiple processes and various cytophysiological changes. The degradation or breakdown of the ECM through protease is a major step in tumor invasion or migration. This phenomenon causes the separation of the intercellular matrix, promoting cancer cell mobility and eventually leading to metastasis. Among the involved proteases, u-PA has one of the most important roles in cancer invasion and metastasis [4]. Ryu et al. reported that resveratrol reduced glioma cell invasion by downregulating mRNA expression of the u-PA and its receptor (u-PA/uPAR) expression [28]. Clinically, the positive relationships between u-PA and metastasis in patients with HCC have been reported [4], [29]. This study verified that resveratrol has an inhibitory effect on metastasis on the u-PA in HCC cells, similar to our previous research on the compound norcantharidin (NCTD) that inhibits MMP-9 and u-PA expression to possess anti-metastatic effects on HCC [1]. However, the present study’s zymography data indicated that the secreted protein level of MMP-2 from Huh7 cells was quite low. Therefore, we concluded that u-PA is the most important proteases in metastasis of human hepatoma.

Mitogen-activated protein kinases (MAPKs) are a family of serine/threonine kinases, such as Jun-N-terminal kinase (JNK), p38, and extracellular signal-regulated kinase (ERK), which respond to chemical and physical stress by connecting cell-surface receptor responses to the activity of regulatory proteins. Activation of MAPKs is followed by phosphorylation of various cytosolic substrates and is involved in numerous cellular programs, such as cell proliferation, cell differentiation, cell invasion, cell migration, and cell death [19], [30]. Furthermore, resveratrol also inhibited PMA-induced MMP-9 expression with the inhibition of JNK activation and PKC activation [31]. However, research on the pathway about u-PA-mediated anti-metastasis effects of resveratrol on HCC cells is rare. This study shows that resveratrol inhibits the phosphorylation of JNK 1/2, leading to the downregulation of u-PA expression in HCC cells. This is the first time that the anti-metastasis effect resveratrol on HCC cells has been verified.

u-PA gene expression is primarily regulated at the transcriptional, posttranscriptional, and protein levels through the use of activators and inhibitors, as well as cell surface localization. The transcription of u-PA genes is regulated by upstream sequences, including motifs that correspond to SP-1 binding sites [32]. The activation of the SP-1 downstream of the MAPK or PI3K-Akt pathways is involved in numerous pathological processes, such as inflammation, cancer-cell adhesion, tumor invasion, metastasis, and angiogenesis [23], [33]. Hsu et al. reported on the u-PA upregulation by stromal cell-derived factor-1 through ERK and JNK phosphorylation with the modulation of the transcription factors SP-1 and AP-1 activity [34]. In this study, we found that resveratrol inhibited the binding activity of SP-1 to the u-PA promoter in Huh7 cells. SP-1 is the transcription nuclear factors that could promote tumorigenesis and regulation and are linked to invasion and metastasis [35], [36]. Suppression of SP-1 has been effective in the prevention and treatment of cancer. Further investigation is required to confirm the role of other transcription factors in the inhibitory effect of resveratrol on u-PA transcription.

In conclusion, this study has shown that resveratrol exerts an inhibitory effect on several crucial stages of metastasis, including cell invasion and migration, by regulating the activities of metastasis-associated proteases and their natural inhibitors. Based on these results, resveratrol is a powerful natural product for developing preventive and treatment agents for cancer metastasis. Furthermore, we demonstrated that resveratrol can effectively inhibit phosphorylation of JNK 1/2 and SP-1 DNA binding activities, causing a downregulation of u-PA expression to inhibit metastasis. Because of the signal transduction mediators and transcriptional factors involved in the resveratrol anti-metastatic process for the human hepatoma cell line, it might be possible to develop specific mediators to inhibit undesired cell metastasis. Future studies should examine resveratrol in vivo to determine its effectiveness in the treatment of hepatoma invasion and migration.
