# Correction: The Antimetastatic Effects of Resveratrol on Hepatocellular Carcinoma through the Downregulation of a Metastasis-Associated Protease by SP-1 Modulation

**DOI:** 10.1371/journal.pone.0174494

**Published:** 2017-03-20

**Authors:** Chao-Bin Yeh, Ming-Ju Hsieh, Chiao-Wen Lin, Hui-Ling Chiou, Pen-Yuan Lin, Tzy-Yen Chen, Shun-Fa Yang

There are errors in the images for Figs 1C and 6D. Please see the corrected Figs 1 and 6 here.

**Fig 1:**
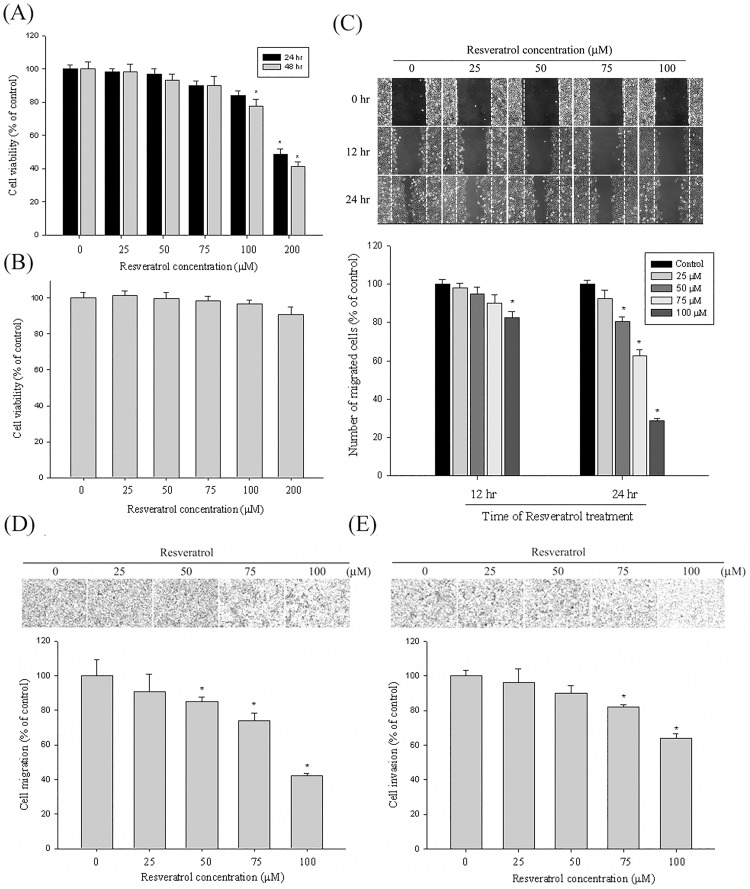
The effect of resveratrol on cell viability, in vitro wound closure, and cell migration and invasion in Huh7 cells. (A) Huh7 cells were treated with resveratrol (0, 25, 50, 75, 100 and 200 μM) for 24 h and 48 h before being subjected to a MTT assay that tested cell viability. The values represented the means ± SD of at least 3 independent experiments. (B) Normal hepatocytes were treated with resveratrol (0, 25, 50, 75, 100 and 200 μM) for 24 h before being subjected to a MTT assay that tested cell viability. The values represented the means ± SD of at least 3 independent experiments. (C) Huh7 cells were wounded and then treated with vehicle (DMSO) or resveratrol (25, 50, 75, and 100 μM) for 0, 12, and 24 h in a 0.5% FBS-containing medium. At 0, 12, and 24 h, the phase-contrast pictures of the wounds at 3 locations were taken. (D & E) Cell migration and invasion were measured using a Boyden chamber for 16 and 24 h with polycarbonate filters, respectively. The migration and invasion abilities of Huh7 cells were quantified by counting the number of cells that invaded the underside of the porous polycarbonate, as described in the Materials and Methods section. The values represented the means ± SD of at least 3 independent experiments. *p<0.05, compared with the vehicle group.

**Fig 6:**
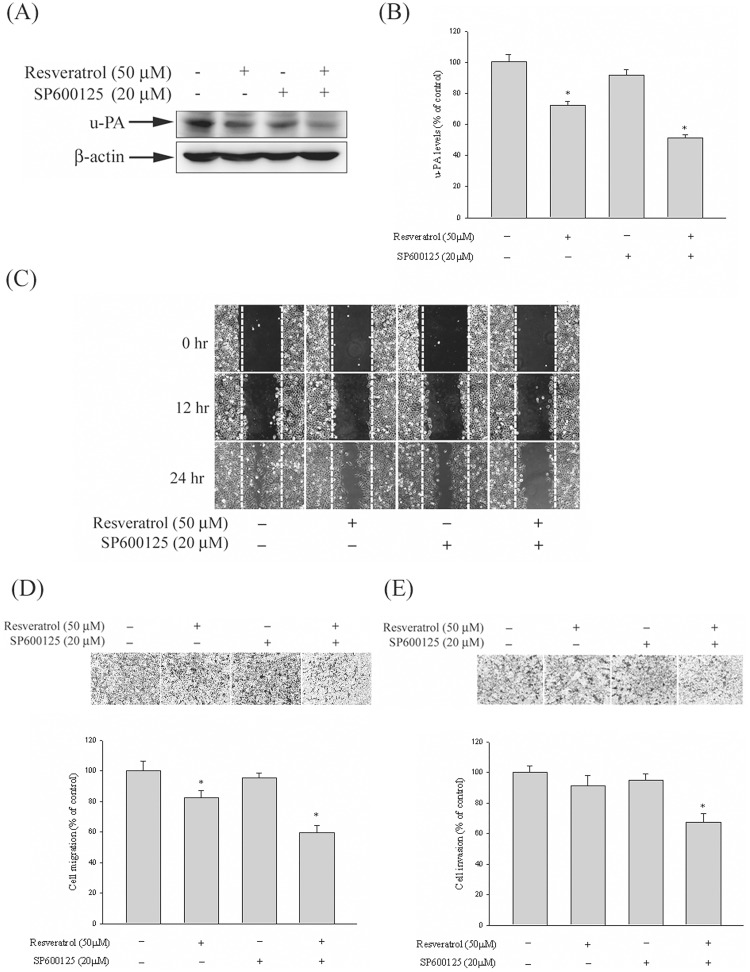
Effect of resveratrol and JNK inhibitor (SP600125) on u-PA expression, *in vitro* wound closure, cell migration and invasion in Huh7 cells. (A–B) Huh7 cells were pre-treated with SP600125 for 30 min and then incubated in the presence or absence of resveratrol for 24 h, and then the cell lysates were subjected to SDS–PAGE followed by western blots with anti-u-PA antibodies as described in Materials and Methods. (C–E) Huh7 cells were pre-treated with SP600125 for 30 min and then incubated in the presence or absence of resveratrol for 24, Huh7 cells were then subjected to in vitro wound closure, cell migration and invasion assay. The migration and invasion abilities of Huh7 cells were quantified by counting the number of cells that invaded to the underside of the porous polycarbonate as described in the Materials and Methods section. The values represented the means ± SD of at least three independent experiments. *p<0.05 as compared with the vehicle group.
