# Expression of Concern: The Antimetastatic Effects of Resveratrol on Hepatocellular Carcinoma through the Downregulation of a Metastasis-Associated Protease by SP-1 Modulation

**DOI:** 10.1371/journal.pone.0306742

**Published:** 2024-07-03

**Authors:** The PLOS ONE Editors

Following the publication and subsequent correction of this article [1, 2], additional concerns were raised regarding results presented in Figure 1. Specifically, the Figure 1E 25μM Resveratrol panel and the Figure 1E 50μM Resveratrol panel appear to partially overlap when one of the panels is flipped horizontally and rotated.

Corresponding author SFY commented that the Figure 1E 25μM Resveratrol panel was misplaced, and provided a replacement figure, the individual-level data underlying the Figure 1 results, and the triplicate image data used in the quantification of the Figure 1D and Figure 1E results (S1 and S2 Files). However, during the assessment of these triplicate image data, additional partial panel overlap was detected in the underlying data between one of the repeats used to quantify the Figure 1D 75μM Resveratrol results and one of the repeats used to quantify the Figure 1D 100μM Resveratrol results. The additional overlap in the image data used for the quantification of the Figure 1D results raises additional questions about the reliability of these data.

The authors did not clarify the availability of the remaining data underlying the published results.

In light of the above concerns, the *PLOS ONE* Editors issue this Expression of Concern to notify readers of these issues, to relay the available data provided by the corresponding author, and to inform readers that the Figure 1 results should be interpreted with caution.

## Supporting information

S1 File Individual-level data underlying results presented in Figure 1. (XLSX)

S2 File Triplicate repeat results used for the quantification of results presented in Figure 1. (ZIP)

## References

[1] Yeh C-B, Hsieh M-J, Lin C-W, Chiou H-L, Lin P-Y, Chen T-Y, et al. (2013) The Antimetastatic Effects of Resveratrol on Hepatocellular Carcinoma through the Downregulation of a Metastasis-Associated Protease by SP-1 Modulation. PLoS ONE 8(2): e56661. doi: 10.1371/journal.pone.0056661 23437203 PMC3577687

[2] Yeh C-B, Hsieh M-J, Lin C-W, Chiou H-L, Lin P-Y, Chen T-Y, et al. (2017) Correction: The Antimetastatic Effects of Resveratrol on Hepatocellular Carcinoma through the Downregulation of a Metastasis-Associated Protease by SP-1 Modulation. PLoS ONE 12(3): e0174494. doi: 10.1371/journal.pone.0174494 28319146 PMC5358885

