# Supplementary figures and images for: Expression of Concern: The Antimetastatic Effects of Resveratrol on Hepatocellular Carcinoma through the Downregulation of a Metastasis-Associated Protease by SP-1 Modulation
Source: PLoS One. 2024 Jul 3;19(7):e0306742. doi: 10.1371/journal.pone.0306742 (PMC11221689; doi:10.1371/journal.pone.0306742)

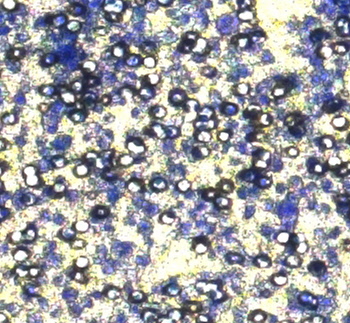

Supplement: S2 File — (ZIP) [file pone.0306742.s002.zip › S2 File/F1D-0-rep 1.jpg]

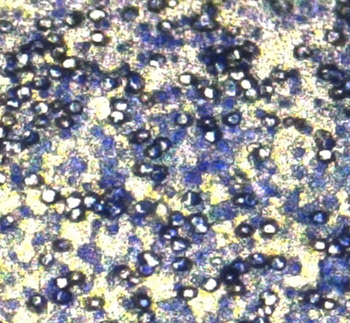

Supplement: S2 File — (ZIP) [file pone.0306742.s002.zip › S2 File/F1D-0-rep 2.jpg]

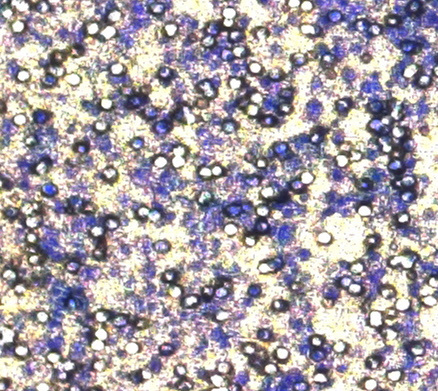

Supplement: S2 File — (ZIP) [file pone.0306742.s002.zip › S2 File/F1D-0-rep 3.jpg]

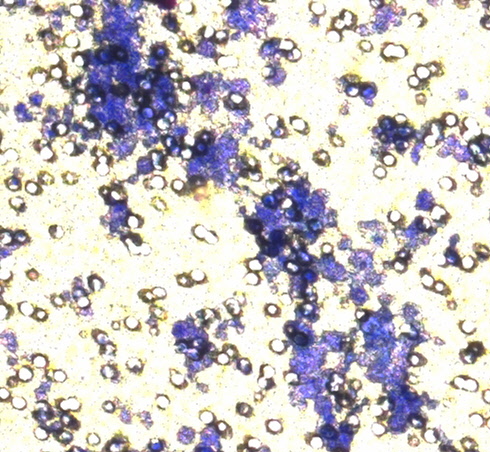

Supplement: S2 File — (ZIP) [file pone.0306742.s002.zip › S2 File/F1D-100-rep 1.jpg]

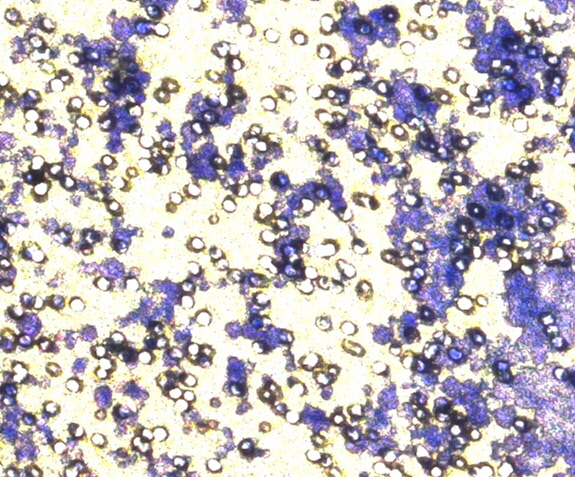

Supplement: S2 File — (ZIP) [file pone.0306742.s002.zip › S2 File/F1D-100-rep 2.jpg]

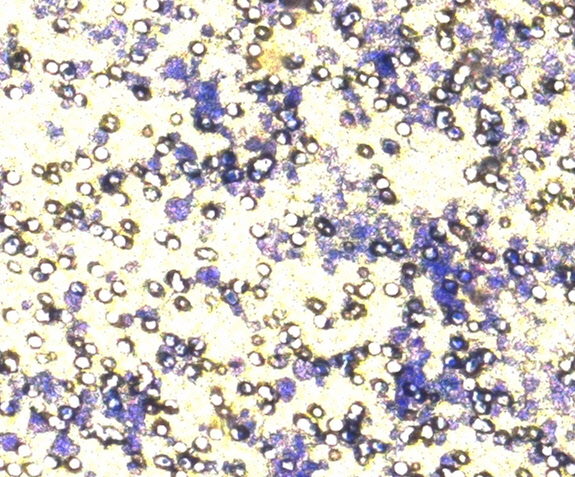

Supplement: S2 File — (ZIP) [file pone.0306742.s002.zip › S2 File/F1D-100-rep 3.jpg]

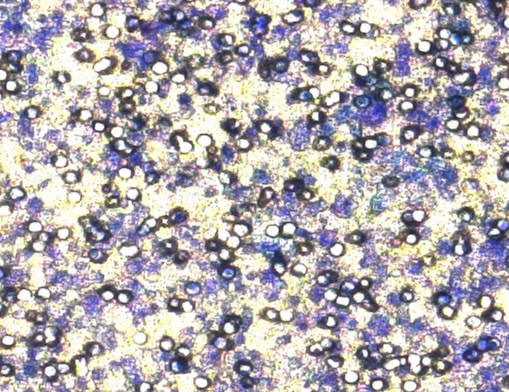

Supplement: S2 File — (ZIP) [file pone.0306742.s002.zip › S2 File/F1D-25-rep 1.jpg]

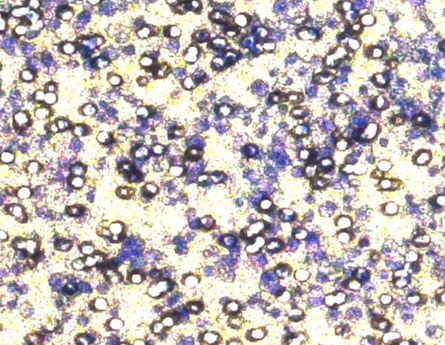

Supplement: S2 File — (ZIP) [file pone.0306742.s002.zip › S2 File/F1D-25-rep 2.jpg]

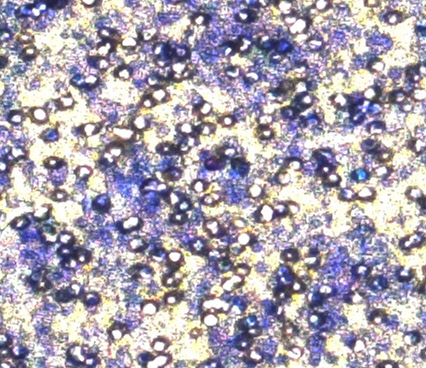

Supplement: S2 File — (ZIP) [file pone.0306742.s002.zip › S2 File/F1D-25-rep 3.jpg]

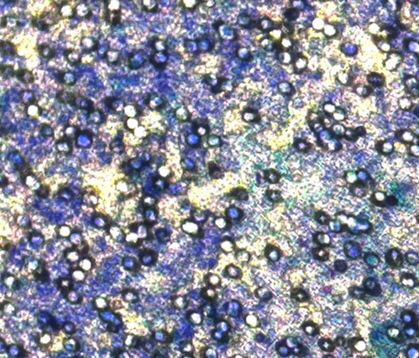

Supplement: S2 File — (ZIP) [file pone.0306742.s002.zip › S2 File/F1D-50-rep 1.jpg]

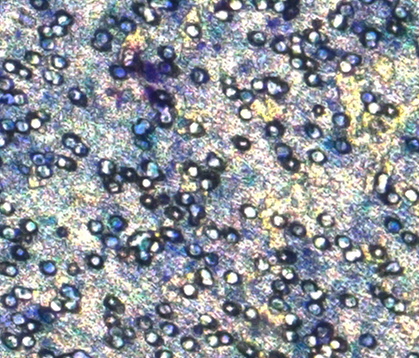

Supplement: S2 File — (ZIP) [file pone.0306742.s002.zip › S2 File/F1D-50-rep 2.jpg]

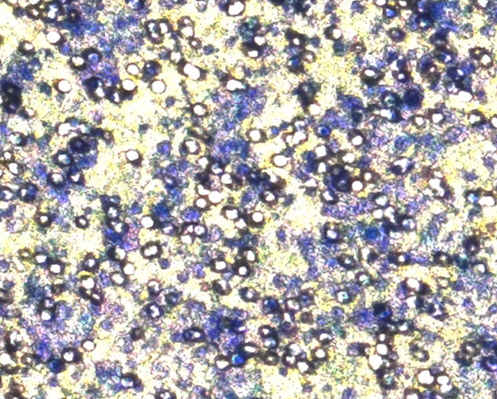

Supplement: S2 File — (ZIP) [file pone.0306742.s002.zip › S2 File/F1D-50-rep 3.jpg]

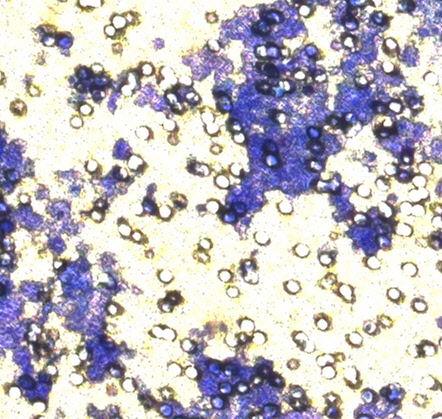

Supplement: S2 File — (ZIP) [file pone.0306742.s002.zip › S2 File/F1D-75-rep 1.jpg]

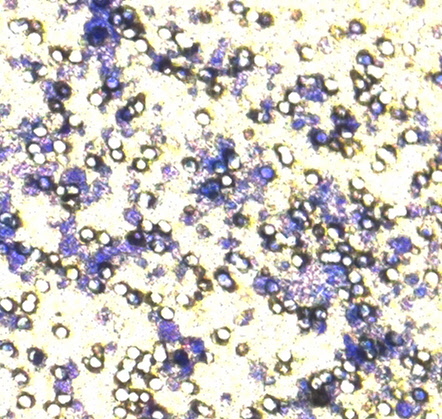

Supplement: S2 File — (ZIP) [file pone.0306742.s002.zip › S2 File/F1D-75-rep 2.jpg]

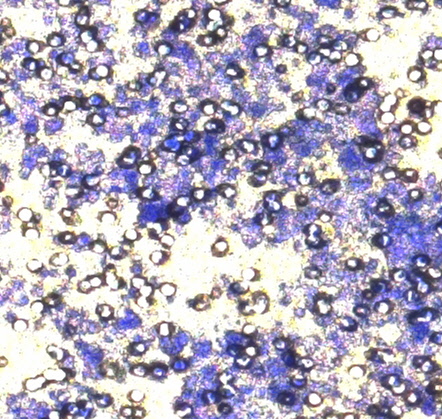

Supplement: S2 File — (ZIP) [file pone.0306742.s002.zip › S2 File/F1D-75-rep 3.jpg]

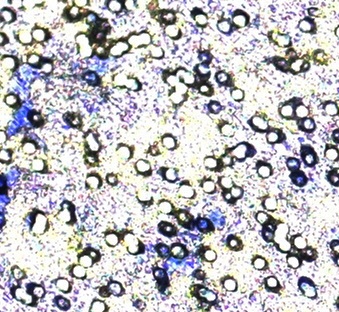

Supplement: S2 File — (ZIP) [file pone.0306742.s002.zip › S2 File/F1E-0-rep 1.jpg]

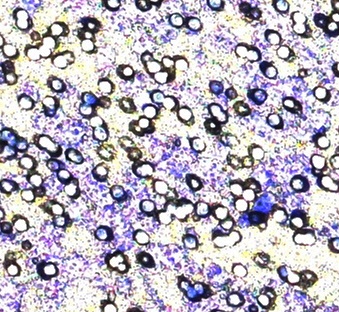

Supplement: S2 File — (ZIP) [file pone.0306742.s002.zip › S2 File/F1E-0-rep 2.jpg]

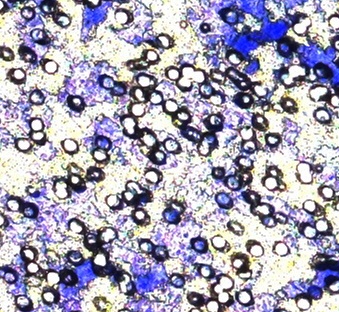

Supplement: S2 File — (ZIP) [file pone.0306742.s002.zip › S2 File/F1E-0-rep 3.jpg]

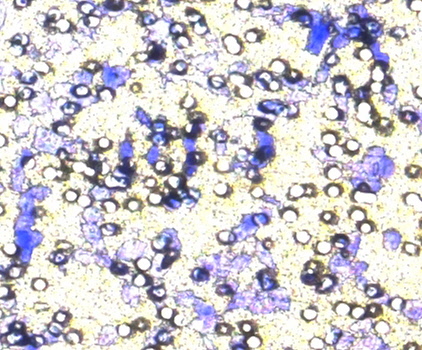

Supplement: S2 File — (ZIP) [file pone.0306742.s002.zip › S2 File/F1E-100-rep 1.jpg]

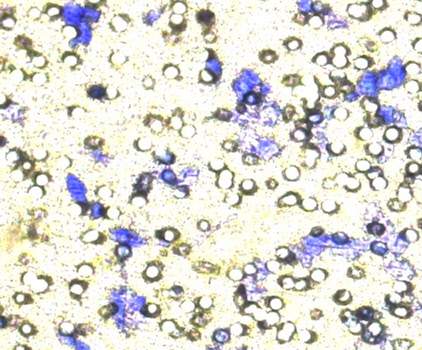

Supplement: S2 File — (ZIP) [file pone.0306742.s002.zip › S2 File/F1E-100-rep 2.jpg]

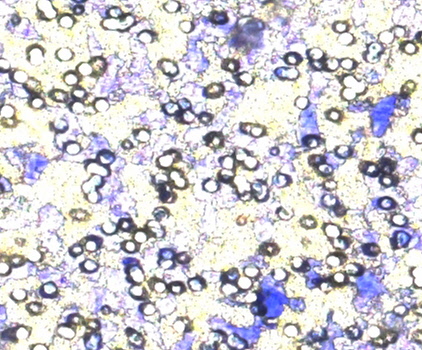

Supplement: S2 File — (ZIP) [file pone.0306742.s002.zip › S2 File/F1E-100-rep 3.jpg]

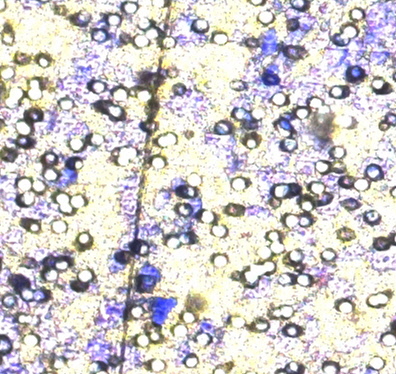

Supplement: S2 File — (ZIP) [file pone.0306742.s002.zip › S2 File/F1E-25-rep 1.jpg]

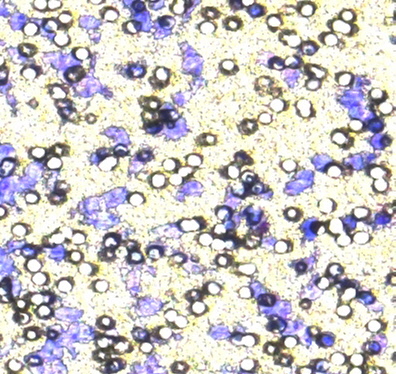

Supplement: S2 File — (ZIP) [file pone.0306742.s002.zip › S2 File/F1E-25-rep 2.jpg]

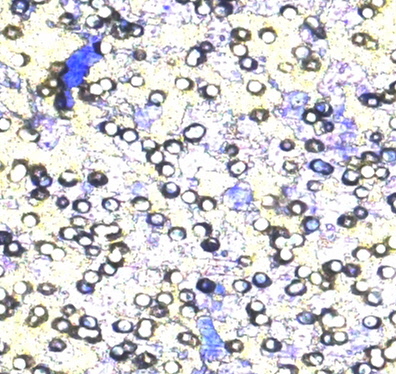

Supplement: S2 File — (ZIP) [file pone.0306742.s002.zip › S2 File/F1E-25-rep 3.jpg]

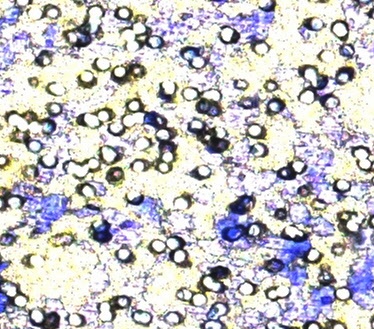

Supplement: S2 File — (ZIP) [file pone.0306742.s002.zip › S2 File/F1E-50-rep 1.jpg]

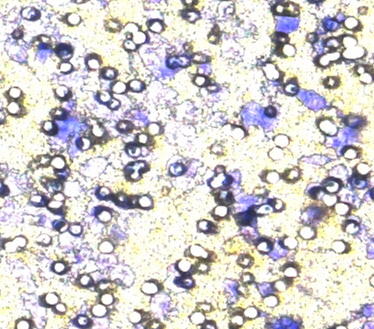

Supplement: S2 File — (ZIP) [file pone.0306742.s002.zip › S2 File/F1E-50-rep 2.jpg]

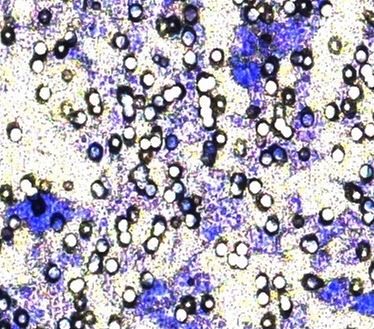

Supplement: S2 File — (ZIP) [file pone.0306742.s002.zip › S2 File/F1E-50-rep 3.jpg]

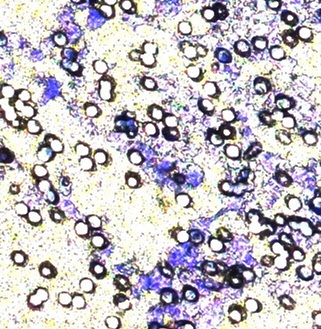

Supplement: S2 File — (ZIP) [file pone.0306742.s002.zip › S2 File/F1E-75-rep 1.jpg]

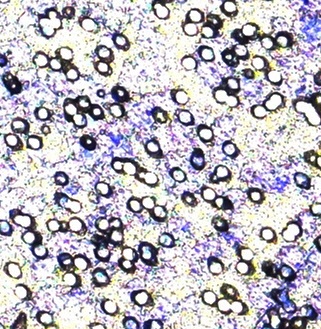

Supplement: S2 File — (ZIP) [file pone.0306742.s002.zip › S2 File/F1E-75-rep 2.jpg]

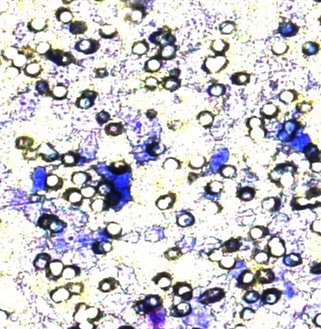

Supplement: S2 File — (ZIP) [file pone.0306742.s002.zip › S2 File/F1E-75-rep 3.jpg]
